# Rare Case of a Transverse Colon Schwannoma

**DOI:** 10.7759/cureus.7604

**Published:** 2020-04-09

**Authors:** Mridul Pansari, Daud Lodin, Anupam K Gupta, Thomas Genuit, Jordan Moseson

**Affiliations:** 1 Surgery, Charles E. Schmidt College of Medicine, Florida Atlantic University, Boca Raton, USA

**Keywords:** schwannoma, laparoscopic surgery, pathology, colon, hemicolectomy, tumor, transverse colon, surgery, colorectal surgery

## Abstract

Schwannomas are tumors comprised of schwann cells of the peripheral nervous system and infrequently present in the gastrointestinal tract. Transverse colon schwannomas are an even more rare subtype of gastrointestinal schwannomas. This study presents the case of a transverse colon schwannoma, in addition to presenting a literature review.

## Introduction

Schwannomas are the tumors originating from schwann cells and their presentation as a gastrointestinal (GI) tumor is very rare, making up a small minority of all GI tumors [1]. Along the GI tract, schwannomas are most commonly found in the stomach and present incidentally during endoscopic examination or computer tomography (CT) scans [2-5]. Colorectal schwannomas are even rarer and patients are typically asymptomatic, but can present with signs of obstruction and rectal bleeding [5].

This case report discusses the findings of a transverse colon schwannoma and discusses the epidemiology, histopathology, differential diagnoses, and treatment of this rare tumor.

## Case presentation

**Figure 1:**
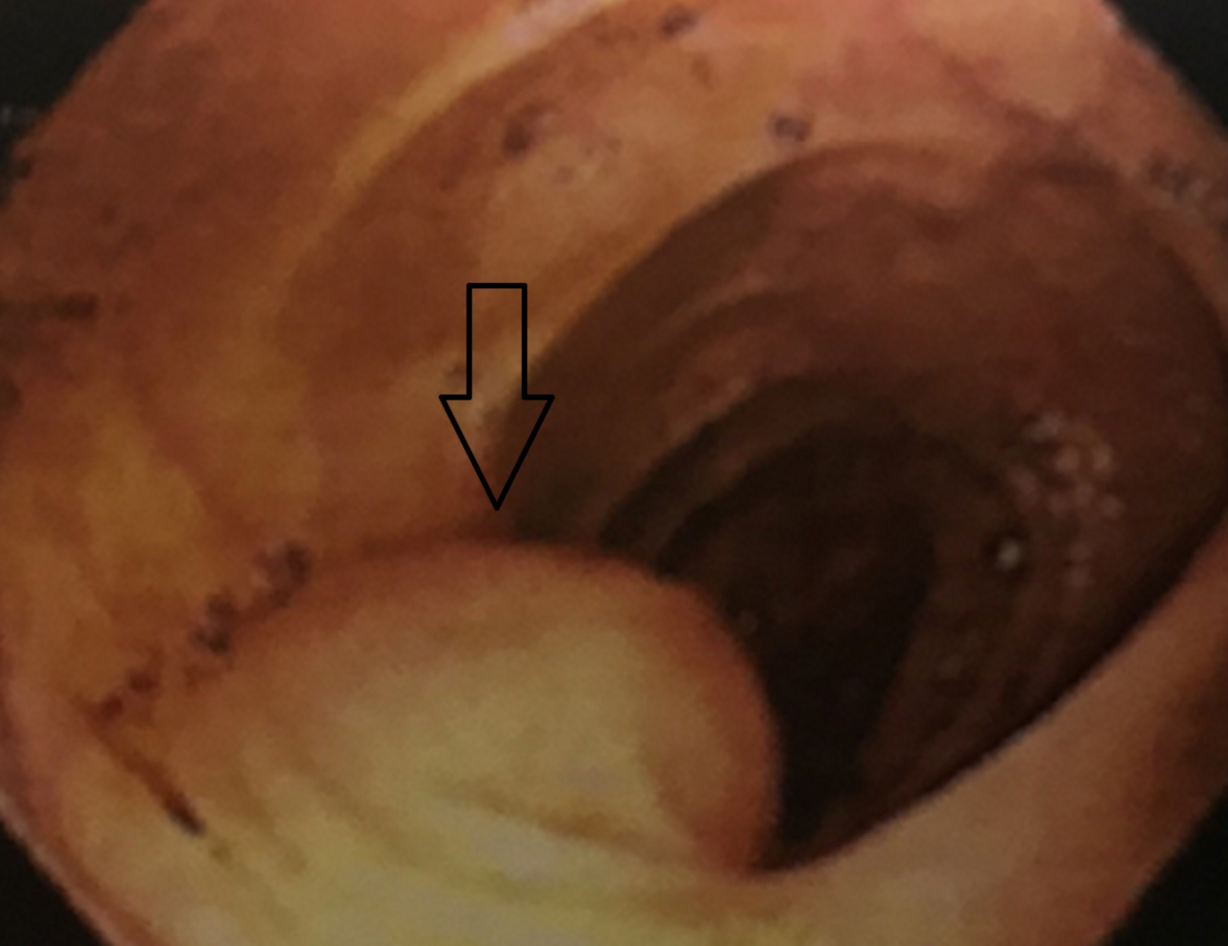
Colonoscopic view of a submucosal mass in the transverse colon.

The patient is a 64-year-old female with no significant past medical history, who underwent age appropriate screening colonoscopy and was found to have a submucosal lesion in the transverse colon (Figure 1).

**Figure 2:**
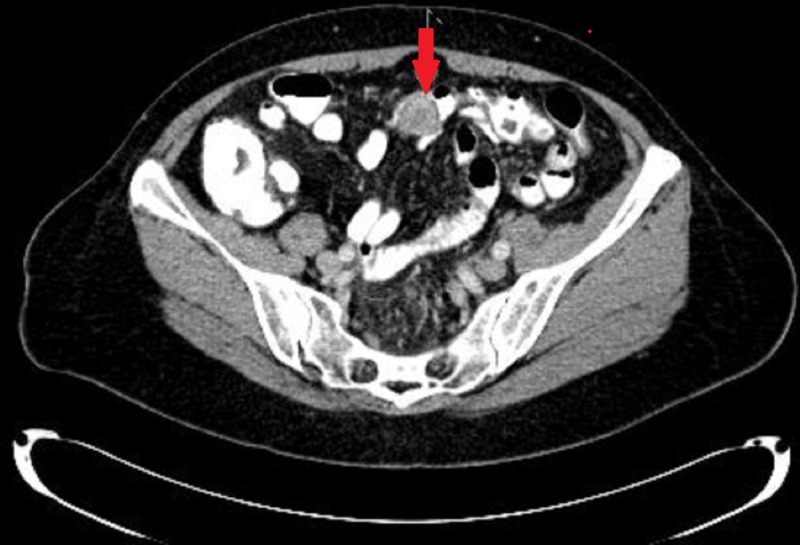
Computer tomography scan of the abdomen, indicating a transverse colon mass 2.0 x 2.1 cm in size.

The mass was tattooed and biopsied. Pathological analysis was unable to diagnose the mass. The patient underwent a computed tomography (CT) scan of her abdomen and pelvis, which demonstrated a 2.1-cm mass in the transverse colon (Figure 2).

**Figure 3:**
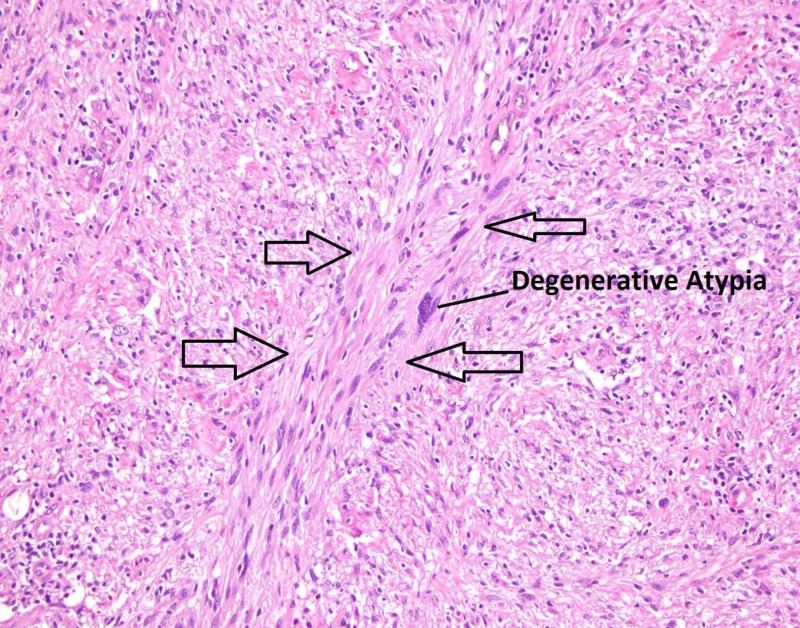
Pathology slide of tumor with haematoxylin and eosin staining at 20x magnification. The arrows point to areas of dense spindle cells with interlacing fascicles. The blue area of density at the center of this image is significant for degenerative atypia, which is associated with schwannomas.

**Figure 4:**
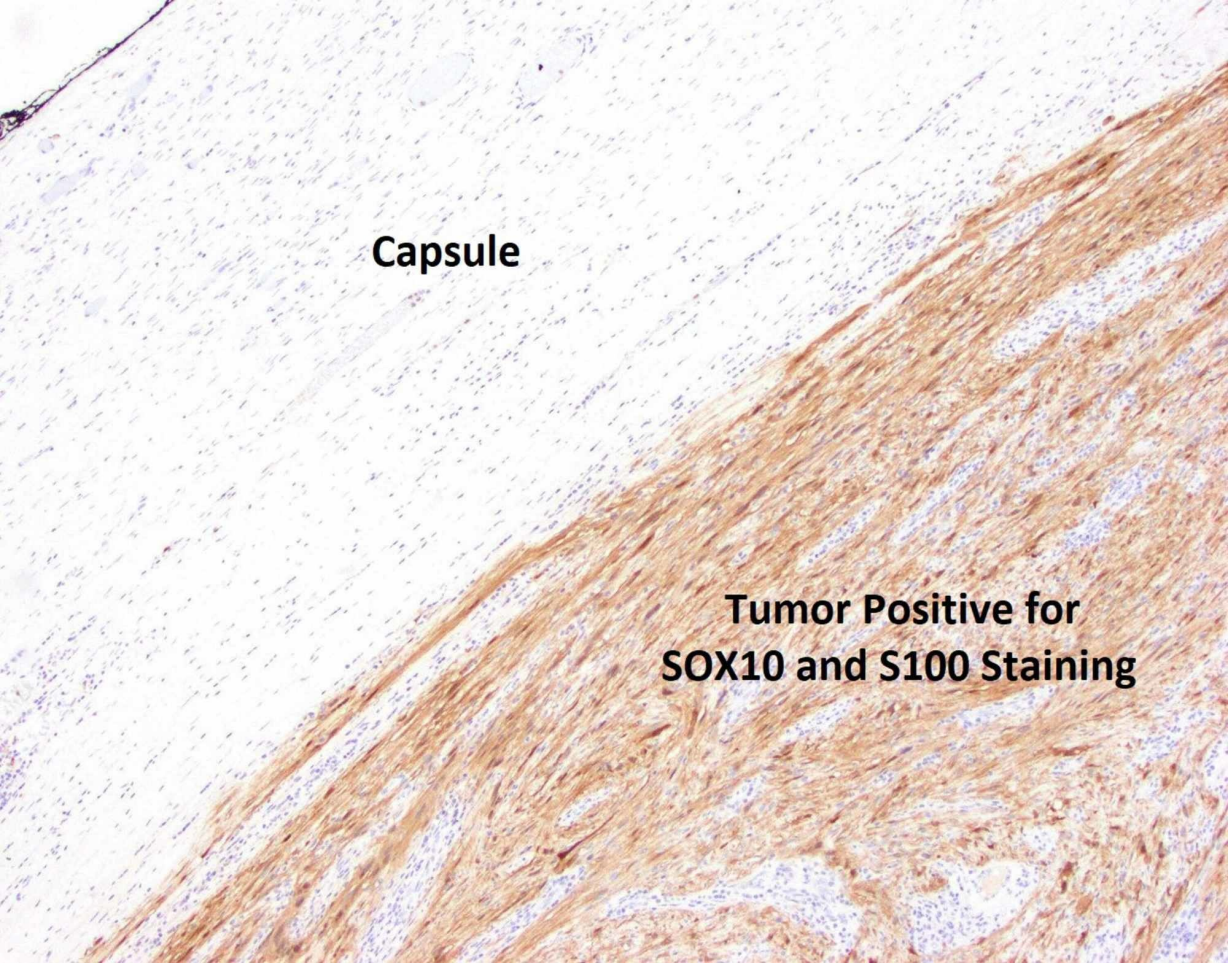
Pathology slide of tumor and capsule with SOX10 and S100 positive staining at 4x magnification. Note that the SOX10 and S100 staining appear dark brown in consistency.

**Figure 5:**
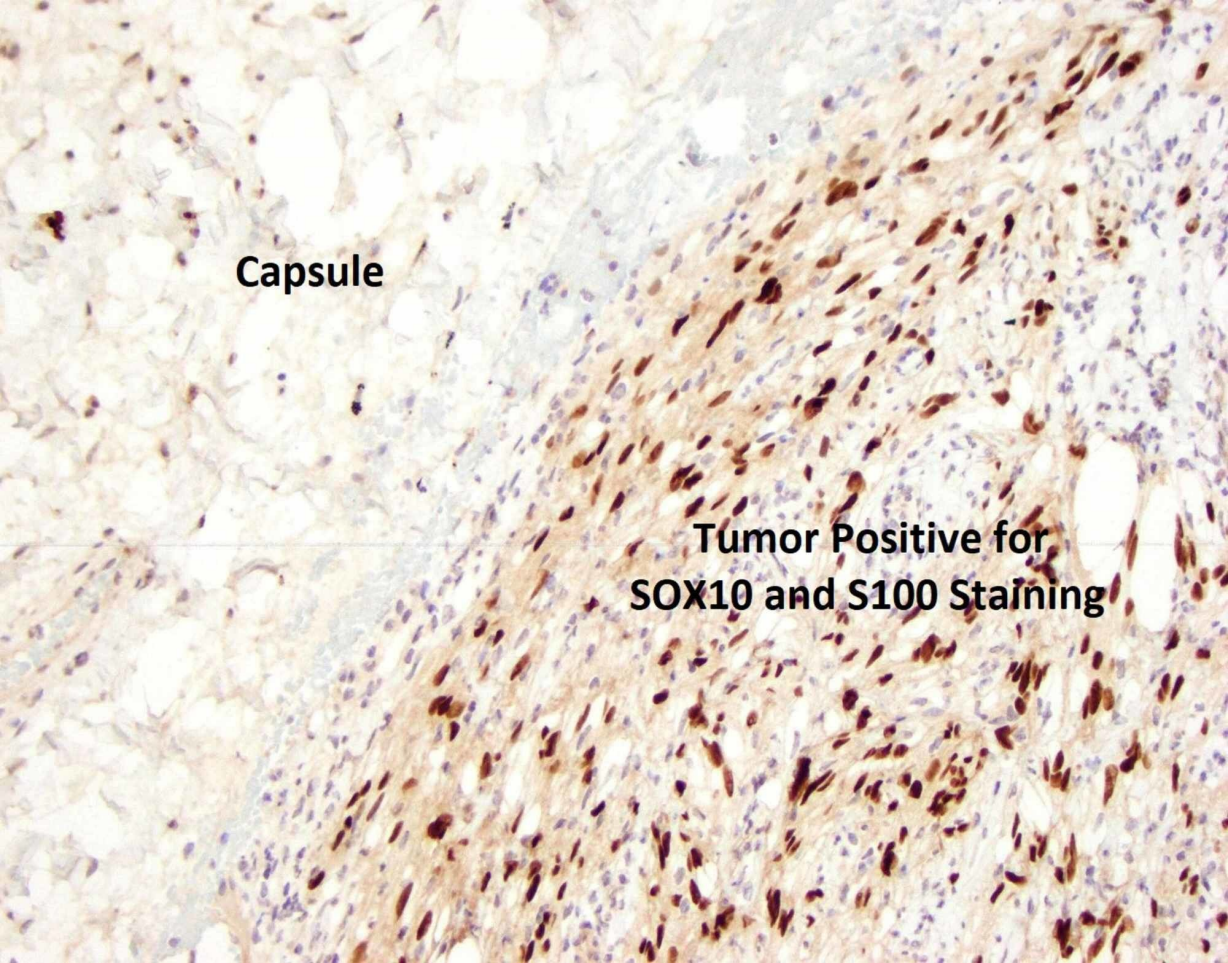
Pathology slide of tumor with SOX10 and S100 positive staining at 20x magnification.

**Figure 6:**
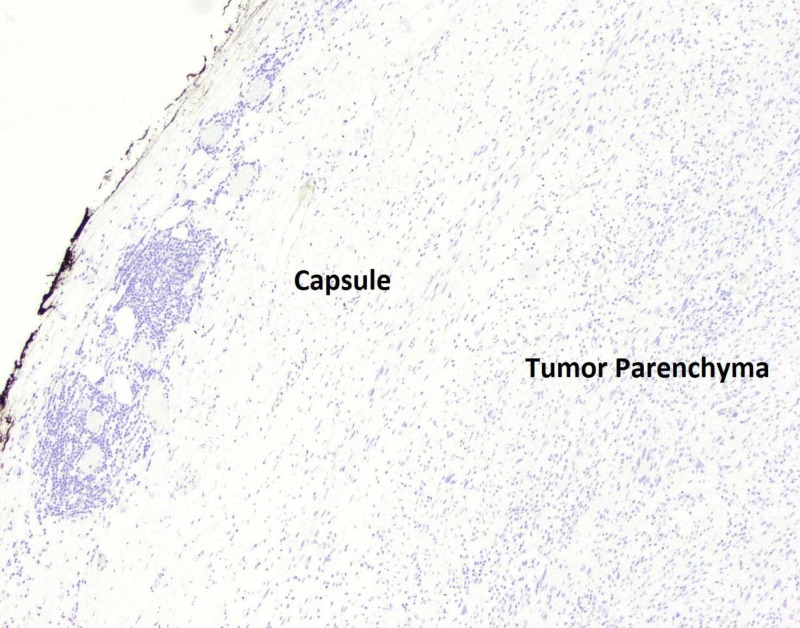
Pathology slide of tumor at the capsular edge with DOG1 negative staining at 4x magnification. Note that standard DOG1 staining will show vibrantly brown and orange in appearance.

**Figure 7:**
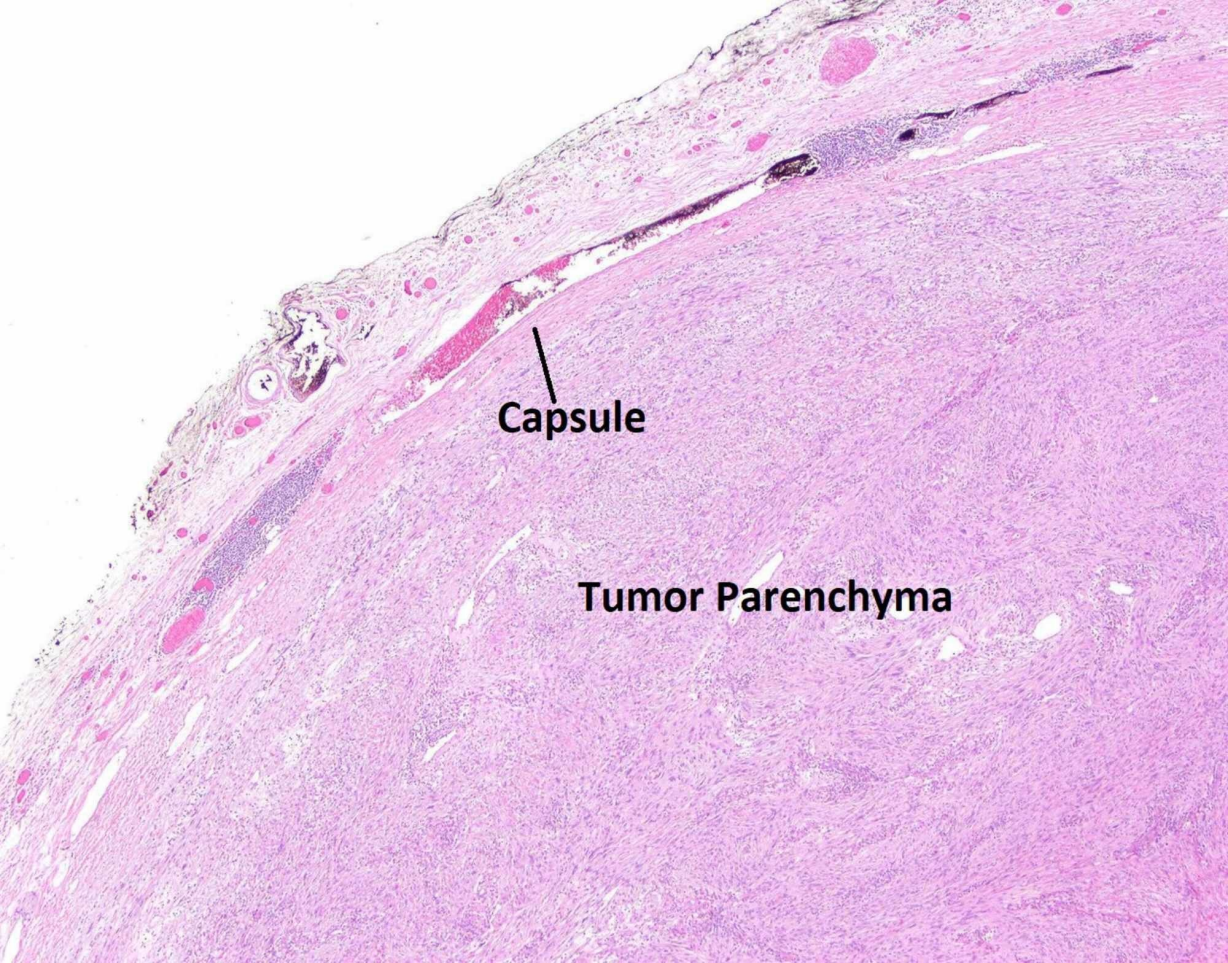
Pathology slide of tumor with haematoxylin and eosin staining at 4x magnification. Note that the tumor does not invade into the capsule, which was consistently noted in all other slides examined.

The patient was scheduled for a laparoscopic extended right hemicolectomy. The procedure was completed successfully with the resection of the right colon and partial resection of the transverse colon with a side-to-side, functional end-to-end terminal ileum to transverse colon anastomosis. The patient had an uneventful postoperative course and was discharged on postoperative day 3 after she tolerated a regular diet. She was seen in clinic two weeks following surgery at which time she denied any complaints. The final pathology report revealed a spindle cell tumor (Figure 3) that stained positive for SOX10 and S-100 (Figures 4, 5) and negative for DOG1, SMA, or CD 117 (Figure 6), consistent with a diagnosis of schwannoma. The Ki-67 index was reported to be 1-2%, which would identify this as a benign lesion. The tumor's capsule was intact without evidence of invasion of the tumor (Figure 7).

## Discussion

Schwannomas are tumors derived from schwann cells. GI schwannomas are very rare and account for approximately 5% of all mesenchymal tumors. GI schwannomas are more often found within the Auerbach’s myenteric plexus and less likely to be found in the Meissner’s submucosal plexus [1]. Mostly commonly found in the stomach, GI schwannomas are less frequently found in the rectum, colon, small intestine, or esophagus [4,5]. Within the colorectal tract, schwannomas typically present in the cecum, right colon, and sigmoid with relatively equally found frequency. Transverse colon schwannomas were found in only 5% of literature cases of all colorectal schwannomas [5].

In a systematic literature review of schwannomas of the colon and rectum published by Bohlok et al., colorectal schwannomas occurred slightly more often in female patients (58%) and had a mean age of onset over the age of 60 years. In their 95-patient study examining colonic or rectal schwannoma, a large portion of patients were asymptomatic on diagnosis (36%), but others presented with rectorrhagia, abdominal pain, or constipation [5]. Other studies noted that GI schwannomas can manifest as colonic obstructions or as intussusception [6-8].

Most GI schwannomas are incidental findings during screening colonoscopy or imaging. During colonoscopy, an incidentally found GI schwannoma appears as a submucosal mass whose overlying mucosa is either smooth in appearance or has an ulceration over the mass [3-5]. When found with CT imaging, it appears as an exophytic mass with homogenous enhancement. Cystic changes, necrosis, or calcifications within tumors are not commonly found [4]. A preoperative diagnosis is challenging, as endoscopic mucosal biopsy of these lesion provides limited incite that could help differentiate these masses from other mesenchymal tumors of the GI tract such as gastrointestinal stromal tumors (GIST), neuroendocrine tumors, leiomyomas, or leiomyosarcomas [5,9]. GISTs are the most common differential diagnosis [10].

A definitive diagnosis is made on immunohistopathological examination of the resected specimen. Macroscopic examination of these tumors will show a well-defined tumor with many lobule appearing areas (Figure 7) that may have an ulceration in their mucosa [11]. Schwannomas stain positive for S100 (Figures 4, 5) and infrequently for vimentin. They stain negative for several markers that help differentiate these tumors from other mesenchymal cells such as DOG1 (Figure 6), SMA, desmin, CD 117, p53, CD-34 and c-kit [10, 12]. Most GISTs are S-100 negative, but CD-34 and c-kit positive [10, 13-16].

Microscopically, schwannomas consist of sheets of fusiform cells with little nuclear pigmentation and a low rate of mitosis (Figure 3). Besides simple microscopic examination and protein staining, pathologists rely on Antoni A and Antoni B classifications, two histological growth patterns that schwannomas demonstrate [2]. Named after the Swedish neuropathologist, Nils Ragnar Eugene Antoni, this system characterizes the two most common forms of growth that schwannomas display. Antoni A histology notes Verocay bodies, fusiform cells densely packed in a palisade formation. Antoni B histology shows a looser group of fusiform cells with oblong or round nuclei seated in xanthomatous histiocytes and myoid stroma [2]. The tumor in this case study was likely Antoni A due to its dense formation of fusiform cells.

Ninety-eight percent of colorectal schwannomas are benign [5]. Two histopathological markers of importance are the Ki-67 index and the mitotic index, both of which are strong predictors for malignancy. A Ki-67 index greater than 5% can correlate to malignancy and any tumor with greater than 10% is considered malignant. A mitotic index of greater than 5 mitotic figures per high power fields is also indicative of a risk for malignancy [9-10]. In Bohlok et al.’s systematic review, three out of 93 cases of colorectal schwannomas were malignant [5]. Their study, however, lacked data on mitotic activity, as well as marker studies for Ki-67. In other case reports describing malignant colorectal schwannomas, malignancy was dependent on the size of the tumor and the number of mitoses present in their specimen, in addition to long-term local recurrence and liver metastasis [17-20].

Complete surgical resection with tumor-free margins is believed to be the best therapeutic option [6]. Tumor recurrence is generally owed to incomplete surgical resection and/or inadequate margins. Adjuvant therapies are not commonly recommended, if surgical resection is able to achieve negative margins [9]. Because of its rarity and the challenge of producing a preoperative diagnosis, review of the clinical features of the disease would be of benefit for surgeons and clinicians who encounter colorectal schwannomas.

## Conclusions

Colorectal schwannomas are a very rare form of benign GI schwannomas that affect mostly women and those over the age of 60. Most cases of these schwannomas are incidentally found during screening colonoscopy and present as submucosal masses. Biopsies do not provide an appropriate diagnosis and surgical resection with tumor negative margins is typically needed for diagnosis and treatment. Immunohistochemistry staining, specifically S-100 tumor marker, will best elucidate the appropriate diagnosis of a schwannoma.
